# Dual‐Functional Terahertz Manipulation of EuBa_2_Cu_3_O_7_ Superconductors via Cooper Pair Dynamics

**DOI:** 10.1002/advs.77616

**Published:** 2026-09-08

**Authors:** Zhangshun Li, Meng Yang, Zuanming Jin, Junxue Li, Zhiqiang Lan, Chen Huang, Alexei V. Balakin, Alexander P. Shkurinov, Yan Peng, Yiming Zhu, Songlin Zhuang

**Affiliations:** ^1^ National Key Laboratory of Terahertz Perception and Communication (TPCL) THz Technology Innovation Research Institute THz Spectrum and Imaging Technology Cooperative Innovation Center Shanghai Key Lab of Modern Optical System University of Shanghai for Science and Technology Shanghai China; ^2^ Department of Physics Southern University of Science and Technology Shenzhen China; ^3^ Shanghai Institute of Intelligent Science and Technology Tongji University Shanghai China; ^4^ Faculty of Physics Lomonosov Moscow State University Moscow Russia; ^5^ Branch “ILIT‐Shatura” of KCCP of NRC “Kurchatov Institute,” Moscow Russia

**Keywords:** superconductors, THz electromagnetic interference shielding, THz modulation, THz spectroscopy

## Abstract

High‐performance terahertz (THz) modulation and electromagnetic intrference (EMI) shielding are vital for next‐gen THz photonics, but conventional materials feature unavoidable tradeoffs among modulation depth, switching speed and shielding efficiency. Copper‐based high‐temperature superconductors (HTS), with their unique Cooper pair dynamics and ultrahigh low‐frequency conductivity, offer an ideal platform to address these challenges. Herein, we systematically investigate dual‐functional THz manipulation in a 43 nm EuBa_2_Cu_3_O_7_ (EBCO) superconducting film using polarization‐tunable THz spectroscopy enabled by a spintronic‐metasurface THz emitter. In the superconducting state, EBCO exhibits an outstanding average THz EMI shielding efficiency of 46.81 dB, outperforming most reported materials. Under 800 nm optical excitation, EBCO delivers an ultrahigh modulation amplitude of 113% with a photocarrier recovery lifetime of 19.0 ps, which is 30‐fold higher than that in the normal state. The superior performance originates from photoinduced Cooper pair dissociation and subsequent quasiparticle recombination. Notably, the THz responses of EBCO are chiral‐insensitive under right‐circularly, left‐circularly, and horizontally linearly polarized THz incidence, indicating preserved or weakly broken time‐reversal symmetry in its d‐wave superconductivity. This work demonstrates that EBCO is a promising candidate for high‐performance cryogenic THz modulators and EMI shielding materials, holding great potential for applications in cryogenic quantum computing and high‐fidelity quantum information transmission.

## Graphical abstract

We use a spintronic‐metasurface THz emitter to realize chiral THz generation. Using polarization‐tunable THz pulses, we investigate equilibrium and photoinduced THz responses in a 43 nm thick EuBa_2_Cu_3_O_7_ (EBCO) thin film. The EBCO film achieves 46.81 dB EMI shielding and 113% photoinduced THz modulation with 19.0 ps recovery, enabled by Cooper‐pair dynamics. Chiral‐insensitive responses reveal symmetry‐related superconducting dynamics.

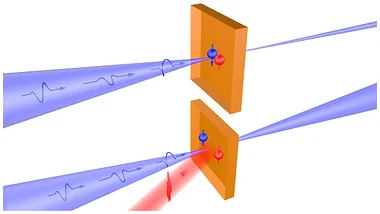

## 1 Introduction

High‐performance ultrafast terahertz (THz) modulators are core components for advancing THz technology [1, 2, 3, 4, 5]. However, common THz modulation materials face a tradeoff between modulation depth (MD) and switching speed [6, 7]: semiconductors have high modulation amplitude but slow switching speed [8, 9], while 2D semimetals enable ultrafast THz responses but suffer from low modulation amplitude [10, 11]. With the rapid development of THz electromagnetic protection and information confidentiality, THz electromagnetic interference (EMI) shielding demand has increased [12], but existing materials lack high efficiency and active tunability [13, 14, 15]. Thus, there is an urgent need for alternative materials integrating efficient THz modulation and ultrafast carrier relaxation to address these issues.

Superconductors feature zero DC resistance and perfect diamagnetism below the critical temperature (*T_c_*), enabling applications such as large‐scale superconducting devices, cryogenic non‐volatile memory, high‐field magnets, and high‐resolution magnetic resonance imaging [16, 17, 18, 19]. The discovery of copper‐based high‐*T_c_* superconductors (HTS) has accelerated superconductor‐based device development, as their superconductivity can be realized above liquid nitrogen temperature [20]. Below *T_c_*, the formation of Cooper pairs endows HTS with extraordinary conductivity [21]. Femtosecond laser excitation can break Cooper pairs on a picosecond timescale, triggering an abrupt transition from superconducting to normal conducting state [22, 23].

Different from conventional superconductors described by BCS theory, cuprate HTS arise from complex coupling among charge, spin, lattice and orbital degrees of freedom [24, 25, 26, 27, 28]. Conventional characterization methods are mostly surface‐sensitive, invasive or limited in probing nonequilibrium competing states [29, 30, 31, 32], while common optical probes employ photon energies far above the superconducting gap and restricting detection of transient Cooper pair dynamics [33, 34, 35]. Notably, THz energy matches the superconducting gap of cuprate HTS [36, 37, 38, 39], offering an ideal tool for detecting low‐energy excitations of Cooper pairs and quasiparticles [40, 41]. Time‐resolved THz spectroscopy has become a powerful technique to unravel intrinsic nonequilibrium physics and collective excitations in cuprate HTS [42, 43, 44, 45, 46, 47]. In addition, the coherent THz emission enables sensitive detection of superconducting fluctuations in cuprate HTS [48, 49, 50]. Beyond fundamental research, cuprate HTS possess great application potential for high‐performance THz modulation and EMI shielding by virtue of their ultrahigh superconducting conductivity and ultrafast photoinduced Cooper pair dynamics. Since quantum computing operates at cryogenic temperatures, superconducting THz manipulation is critical for high‐fidelity quantum information transmission, underscoring the significance of exploring superconducting THz optoelectronic properties. However, symmetry‐governed Cooper pair dynamics and active control of carrier behavior in cuprate HTS remain largely unexplored, constrained by the limited ability of conventional optical components to generate and manipulate chiral THz radiation [51, 52].

Herein, we investigate ultrafast THz modulation in a 43 nm near‐optimally doped EuBa_2_Cu_3_O_7_ (EBCO) film using polarization‐tunable THz spectroscopy. Chiral THz radiation is generated and switched using a spintronic‐metasurface THz emitter (SMTE) with tunable in‐plane magnetic field [53, 54]. In the superconducting state, EBCO exhibits temperature‐dependent THz EMI shielding performance with an average shielding efficiency of 46.81 dB within 0.3–1.0 THz. Under 800 nm photoexcitation, EBCO shows an ultrafast modulation speed (1.7 ps) and a low modulation amplitude (3.5%) in the normal state. Remarkably, the EBCO in the superconducting state delivers a high modulation amplitude (113%) with a 19.0 ps photocarrier recovery lifetime, originating from photoinduced Cooper pair dissociation and subsequent quasiparticle recombination. Furthermore, we demonstrate the dual‐functional THz modulation is chiral‐insensitive, which indicates preserved or weakly broken time‐reversal symmetry in Cooper pair dynamics.

## 2 Results and Discussion

### 2.1 Experiment and Samples

**FIGURE 1:**
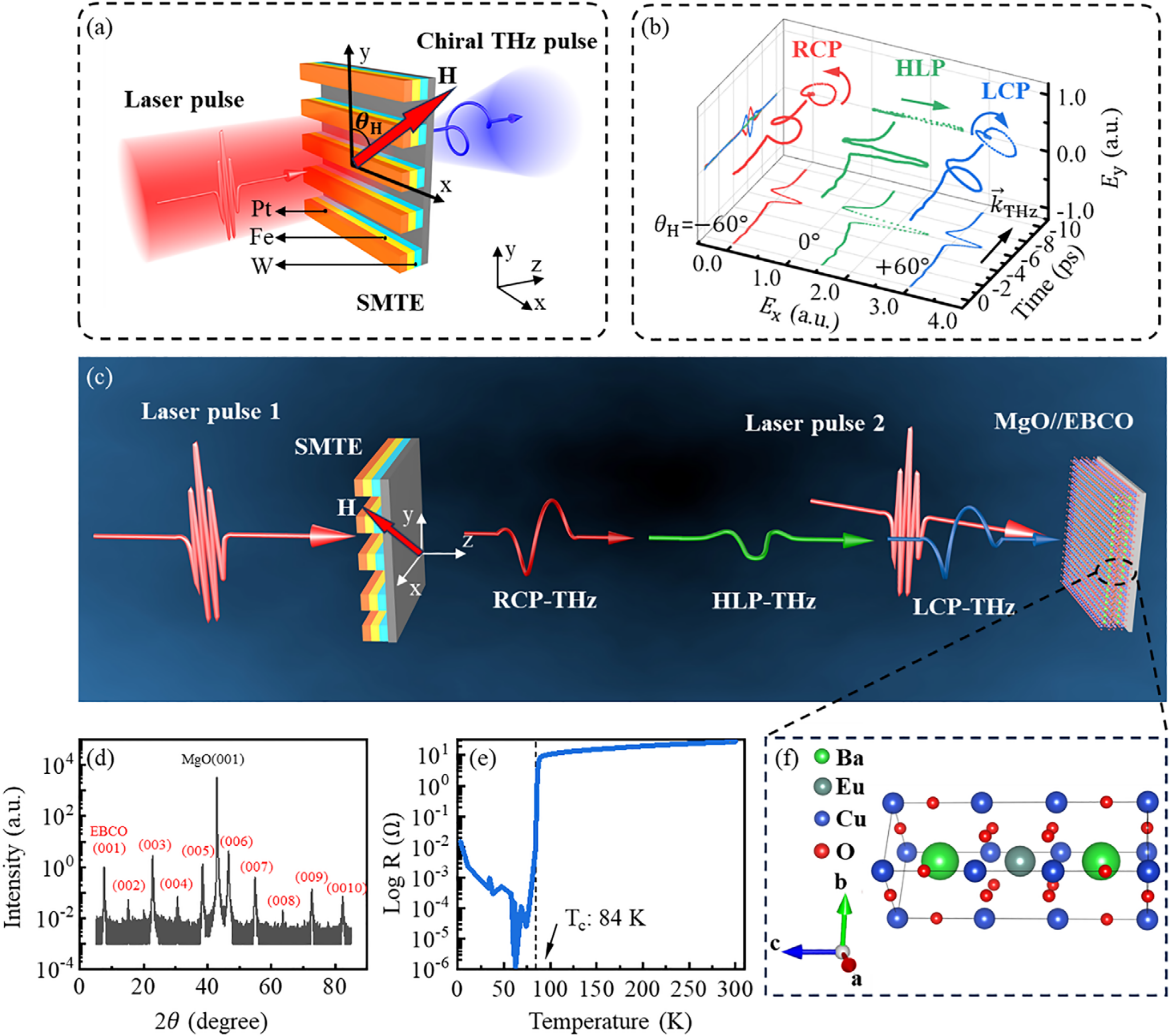
Schematic of polarization‐controllable THz spectroscopy measurement and EuBa_2_Cu_3_O_7_ sample structure. (a) Schematic of polarization‐controllable THz wave emission from a SMTE. (b) RCP, HLP and LCP THz pulse generated by the SMTE at θ_*H*_ = − 60°, 0°, and +60°. (c) Schematic of time‐resolved polarization‐controllable THz spectroscopy measurement. (d) X‐ray diffraction pattern of the 43 nm‐EBCO film. (e) Temperature‐dependent resistance of the 43 nm‐EBCO film. (f) Crystal structure of the EBCO film.

Figure 1a shows the schematic of polarization controllable THz wave emission from the SMTE. Figure 1b shows the time‐domain THz waveforms emitted from the SMTE at θ_*H*_ = − 60°, 0° and +60°, where θ_*H*_ denotes the angle between the magnetic field and the normal direction of the stripe array. Based on the wave vector *k* direction, θ_*H*_ = − 60°, 0° and +60° generate the right‐circularly polarized (RCP), horizontal linearly polarized (HLP) and left‐circularly polarized (LCP) THz pulses, respectively. To ensure experimental reliability, we selected 0.3‐1.0 THz spectral data for analysis, utilizing the over 40 dB dynamic range (Figure S1 and Note S1).

Figure 1c shows the ultrafast polarization‐controllable THz spectroscopy. A Ti: sapphire laser amplifier's output was split into three beams: laser pulse 1 excited the SMTE to generate RCP, HLP, and LCP polarized THz pulses, laser pulse 2 photoexcited the EBCO, and the third detected THz waves transmitted through EBCO via electrooptical sampling (EOS). Equilibrium and non‐equilibrium Cooper pair electrodynamics in EBCO film were probed by switching the pump laser off/on. The EBCO film was placed in a cryostat (20–300 K). Details of the optical pump‐THz probe spectroscopy are provided in Methods.

Our sample is a 43‐nm‐thick EBCO film deposited on a 0.5 mm‐thick single‐crystalline MgO (001) substrate via pulsed‐laser deposition (see **Experimental Section**). Figure 1d shows the XRD peaks of EBCO film, confirming the good crystallinity of sample. Figure 1e shows the zero‐resistance superconducting critical temperature *T_c_* = 84 K of EBCO film, which was obtained by resistance measurement. The EBCO along the *c*‐axis is made of bilayers of CuO_2_ planes, spaced by Cu‐O chains and separated by europium layers, as depicted in Figure 1f.

### 2.2 THz EMI Shielding of the EBCO Film

**FIGURE 2:**
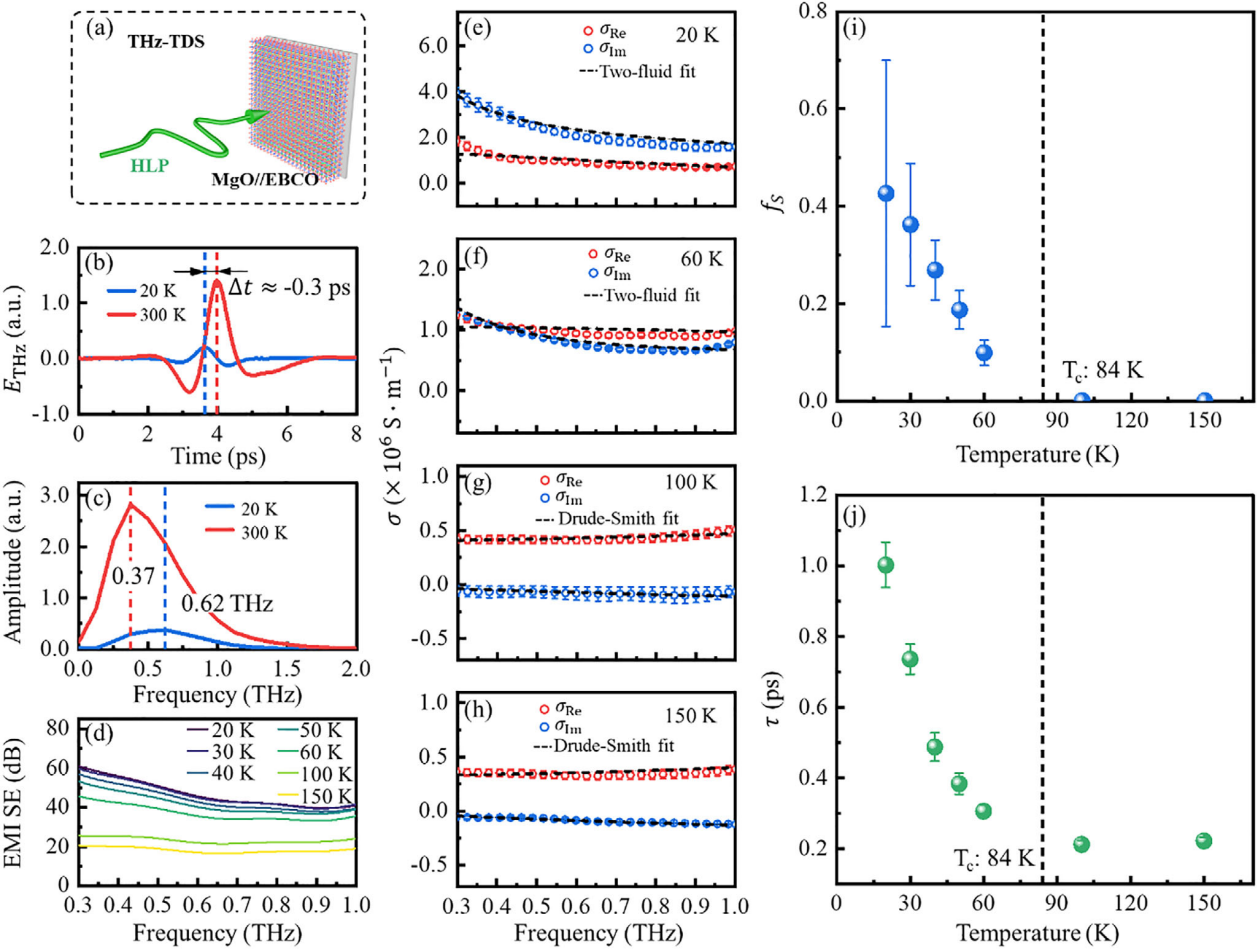
Temperature dependence of THz conductivities measured with HLP THz pulse at equilibrium condition. (a) Schematic of THz time‐domain spectroscopy measurement for the EBCO film. (b) *E*_THz_(*t*) of the EBCO film at 20 and 300 K. (c) Frequency‐domain spectra of the EBCO film measured at 20 and 300 K. (d) THz EMI shielding of EBCO film measured at different temperatures. THz conductivity spectra of EBCO measured at (e) 20 K, (f) 60 K, (g) 100 K, and (h) 150 K. Red and blue circles denote real and imaginary parts of the conductivity spectra. The dashed curves in (e,f) are two‐fluid fit. The dashed curves in (g,h) are Drude‐Smith fit. The error bar represents the uncertainty in the measurements of the THz conductivity. Temperature dependences of (i) fraction of superfluid *f_S_* and (j) momentum scattering time τ. The error bar represents the uncertainty in the fitting of the THz conductivity.

We first investigated the THz EMI shielding properties of the EBCO film. The schematic of THz time‐domain spectroscopy (THz‐TDS) with HLP THz pulses is shown in Figure 2a. Figure 2b shows the time‐domain electric field *E*_THz_(*t*) of the EBCO film measured at 20 and 300 K (*E*_THz_(*t*) measured at other temperatures are provided in Figure S2 and Note S2). The *E*_THz_(*t*) amplitude at 20 K is significantly reduced compared to that at 300 K, with waveform shifted forward by ∼0.3 ps. Corresponding frequency‐domain spectra (Figure 2c) show a distinct blueshift of the spectral center frequency, from 0.37 THz at 300 K to 0.62 THz at 20 K. These behaviors indicate electromagnetic signatures of the superconducting state [42, 55].

EMI shielding describes the attenuation of radiated electromagnetic waves, where shielding efficiency (SE) is defined as [12, 56]:

(1) $$\mathrm{EMI}\;\mathrm{SE}\;=-20\log\left(\frac{E_{\mathrm{sam}}\left(\omega \right)}{E_{\mathrm{sub}}\left(\omega \right)}\right)$$

where *E*_sam_(ω) and *E*_sub_(ω) are the Fourier transforms of *E*_sam_(*t*) and *E*_sub_(*t*) for the EBCO film on MgO substrate and bare MgO substrate, respectively. In Figure 2d shows the temperature dependent EMI SE of the 43‐nm‐thick EBCO film measured for HLP THz pulse. The EMI SE reach up to about 46.81 dB (averaged over the 0.3–1.0 THz) at 20 K. The specific SE (EMI SE/thickness) for EBCO film is 1088.60 dB/µm at 20 K. In the superconducting state, EMI SE is enhanced at low frequencies, originating from Cooper pairs formation and low‐frequency redistribution of conductivity spectral weight. At 0.3 THz and 20 K, the specific SE value reach to 1415.81 dB/µm. The EMI SE of EBCO film measured for RCL and LCP THz pulses are provided in Note S3 (Figure S3). As summarized in Table 1, in the superconducting state, EBCO exhibits higher EMI SE than mostly reported materials, satisfying the requirements for THz EMI shielding. Although copper foil (at room temperature) can achieve EMI SE values comparable to those of EBCO thin films at 20 K, low‐temperature EBCO thin films exhibit unique optically tunable functionalities that static metallic shielding materials such as copper foil cannot offer (see Section 2.3). This renders superconducting EBCO thin films highly promising for applications including low‐temperature THz devices and quantum computing.

**TABLE 1:** Comparison of THz EMI shielding performance for various materials.

Materials	Thickness (µm)	EMI SE (dB)	Specific SE (dB/µm)	Attenuation coefficient (µm^−1^)	Frequency range (THz)	Refs.
PdTe_2_	0.020	19.65	982.50	113.11	0.2–2.0	[12]
Ti_3_C_2_T_y_ film	0.025	2.5	100	11.51	0.25–2.25	[57]
Graphene foam	3000	74	0.025	0.003	0.1–1.6	[58]
Graphitic carbon microspheres	3.75	27.5	7.33	0.84	0.1–1.6	[59]
Al foil	8	66	8.25	0.95	0.008–0.012	[60]
Cu foil	0.04	44.22	1113.75	128.22	0.1–1.0	[61]
Cu/graphene	0.160	60.95	380.9	43.85	0.1–1.0	[61]
EuBa_2_Cu_3_O_7_	0.043	46.81 (@ 20 K)	1088.60 (@ 20 K)	125.33	0.3–1.0	This work
EuBa_2_Cu_3_O_7_	0.043	19.90 (@ 150 K)	462.79 (@ 150 K)	53.28	0.3–1.0	This work

Within the thin film approximation, the complex THz conductivity spectrum $\tilde{\sigma }\left(\omega \right)$ is given by [62]:

(2) $$\frac{E_{\mathrm{sam}}\left(\omega \right)}{E_{\mathrm{sub}}\left(\omega \right)}=\frac{1+n_{\mathrm{sub}}}{1+n_{\mathrm{sub}}+Z_{0}d\tilde{\sigma }\left(\omega \right)}$$

where *Z*_0_ = 377 Ω denotes the free‐space impedance, *n*_sub_ = 3.14 is the refractive index of the MgO substrate in the THz range [63], and *d*  =  43 nm is the thickness of the EBCO film. Figure 2e–h show the real part σ_Re_ and imaginary part σ_Im_ of the complex conductivity $\tilde{\sigma }\left(\omega \right)$ for the EBCO film obtained via HLP THz pulses, measured at 20, 60, 100 and 150 K, respectively. The THz conductivity spectra of EBCO acquired under RCL and LCP THz pulses at different temperatures are present in Note S4 (Figure S4). At 150 K, σ_Re_ is positive and relatively flat across the entire THz frequency range, while σ_Im_ is negative with a small magnitude. The negative σ_Im_ is an intrinsic electronic feature originating from nanoscale inhomogeneous electron scattering within the EBCO film under the normal state. This characteristic has also been observed in certain metallic thin films Mn_2_Au [64]. Below *T_c_* (84 K), σ_Re_ is relatively small, because the low‐frequency spectral weight has condensed into a δ‐function at zero frequency [65, 66]. While, σ_Im_ turns positive and its low‐frequency component increasing drastically (see in Figure S5 and Note S5). For *T* << *T_c_* (e.g. *T* = 20 K), σ_Im_ exhibits a typical 1/ω divergent behavior, which demonstrates that the EBCO film enters the superconducting state [42, 55]. The shift in spectral weight of σ_Im_ toward lower frequencies enhance the low‐frequency THz conductivity of the film, which accounts for both the spectral blueshift observed in Figure 2c and the enhanced low‐frequency EMI SE presented in Figure 2d.

In addition, the forward shift of the transmitted THz time‐domain waveform below *T_c_* can be attributed to the large kinetic inductance contributed by the superconducting Cooper pair condensate. Upon entering the superconducting state, the σ_Im_ rises dramatically, which modifies the effective complex refractive index of the EBCO film and reduces the phase delay accumulated by THz waves propagating through the thin film. For all frequency components composing the single‐cycle THz pulse, the smaller phase shift leads to an earlier arrival time of the overall waveform in the time domain. In contrast, the normal state above *T_c_* exhibits negligible σ_Im_ and larger effective optical path delay, resulting in a delayed THz peak position. This temporal shift is a characteristic inductive electrodynamic fingerprint of cuprate high‐*T_c_* superconductors.

The $\tilde{\sigma }\left(\omega ,T\right)$ of EBCO film above *T_c_* is described by Drude‐Smith mode [64, 67, 68]:

(3) $$\tilde{\sigma }\;\left(\omega ,T\right)=\frac{\varepsilon_{0}\omega_{p}^{2}\left(T\right)\tau \left(T\right)}{1-i\omega \tau \left(T\right)}\;\left(1+\frac{c\left(T\right)}{1-i\omega \tau \left(T\right)}\right)$$

where ε_0_ is the vacuum permittivity, ω_*p*_ is the plasma frequency, τ is the momentum scattering time, and *c* is the carrier scattering parameter ranging from 0 to − 1. By Drude‐Smith model fit, *c* (T = 150 K) ≈ −0.76 and *c* (T = 100 K) ≈ −0.71, which indicates carrier backscattering or electronic inhomogeneities in the normal state.

The $\tilde{\sigma }\left(\omega ,T\right)$ of EBCO film below *T_c_* is described by phenomenological two‐fluid mode [42, 69, 70]:

(4) $$\tilde{\sigma }\;\left(\omega ,T\right)=\frac{n_{\mathrm{QP}}\left(T\right)\tau \left(T\right)}{1-i\omega \tau \left(T\right)}+n_{S}\left(T\right)\left(\pi \delta \left(\omega \right)+\frac{i}{\omega }\right)$$

where *n*_QP_(*T*) and *n*_S_(*T*) denote the effective densities of quasiparticle and superfluid, respectively. $\tilde{\sigma }\left(\omega ,T\right)$ captures the mixed electronic response from the superconducting condensate and normal quasiparticles. σ_Re_ serves as a measure of the quasiparticle fraction, while σ_Im_ as a measure of the superfluid fraction. The sum of *n*_QP_(*T*) and *n*_S_(*T*) is fixed by the total electron number, with an assumed constant. The fraction of superfluid *f_S_* (*T*)  = *n*_S_ (*T*)/(*n*_QP_(*T*) + *n*_S_(*T*)). τ(*T*) and *f_S_* (*T*) are set as free fitting parameters (see reasonable fits in Figure S5). In Figure 2i, the temperature‐dependent *f_S_* (*T*) shows dramatic changes near the *T_c_*. The onset of *f_S_* (*T*) corresponds to the condensation of the majority of charge carriers into Cooper pairs, consistent with *T_c_* determined from temperature‐dependent electric resistivity. At 20 K, the superconducting gap Δ = 20 meV is larger than the photon energy of 1 THz radiation (4.14 meV), thus ensuring the superconducting state remains unperturbed by THz waves. Below *T_c_*, $\tilde{\sigma }\left(\omega \right)$ dominated by the superfluid response, as shown in Figure 2j, τ(*T*) increases drastically, corresponding to a sharp reduction in the carrier scattering rate. This trend aligns with that observed in underdoped or optimally doped Bi‐2212 [39]. Microwave spectroscopy on YBCO crystals also revealed an abrupt rise in τ below *T_c_*, attributed to suppressed thermally excited quasiparticles [55]. The pronounced decrease in the scattering rate can be attributed to the opening of the superconducting gap in EBCO below *T_c_* [71, 72]. In an ideal limit, τ would approach infinity in the pure superconducting state. However, residual resistivity in the EBCO film limits this value to a finite magnitude of τ∼1.00 ±  0.06 ps at 20 K.

**FIGURE 3:**
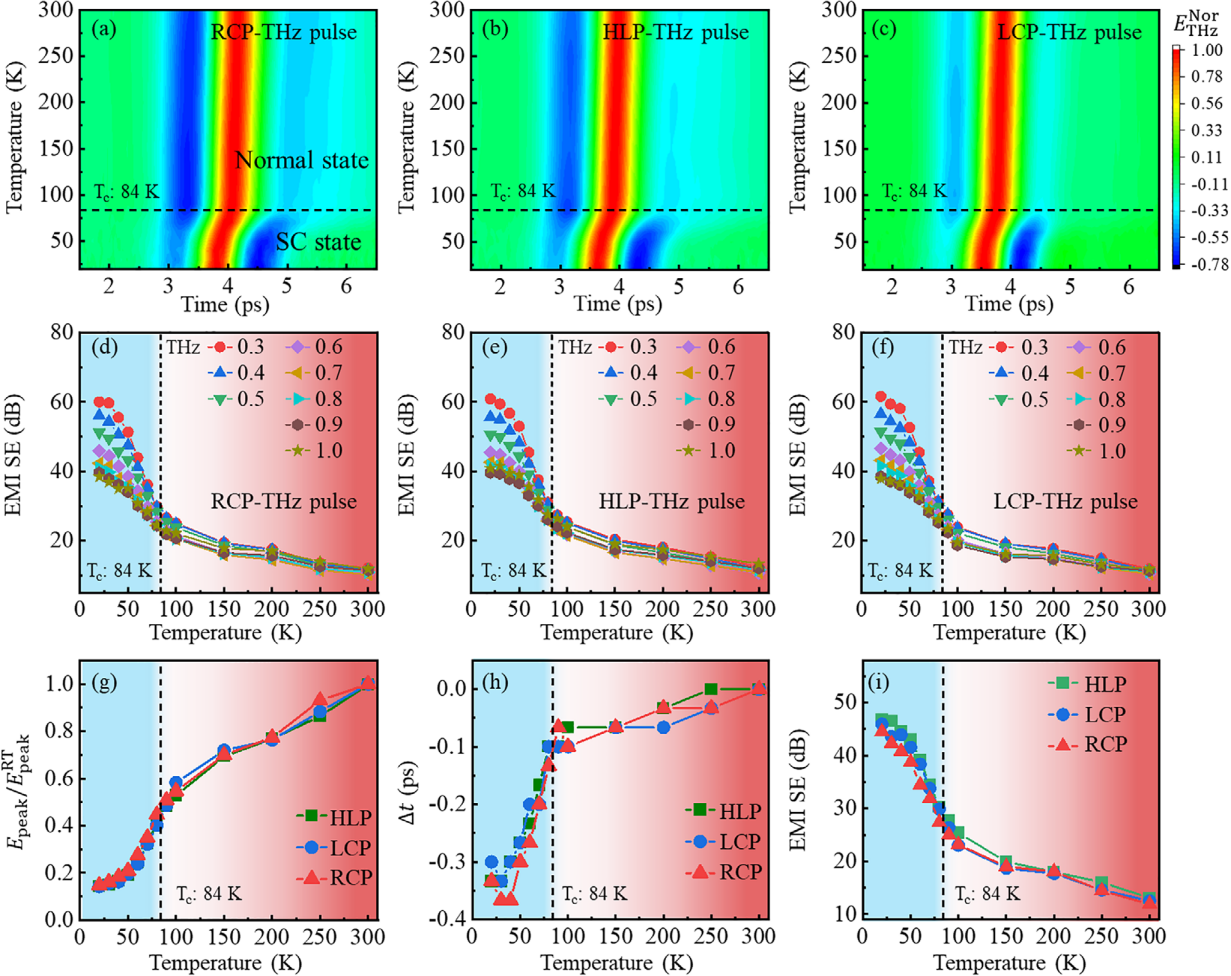
Temperature dependences of THz EMI shielding performance for different polarized THz pulses. Normalized 2D contour maps of THz time domain signals for the 43 nm thick EBCO film, acquired with (a) RCP, (b) HLP and (c) LCP THz excitation under various temperatures. Temperature‐dependent EMI SE as a function of frequency for (d) RCP, (e) HLP and (f) LCP THz pulses. Temperature dependence of (g) normalized peak transmittance $E_{\mathrm{peak}}\left(T\right)/E_{\mathrm{peak}}^{\mathrm{RT}}$, where RT denotes room temperature, (h) peak time shift Δ*t*, and (i) average EMI SE in the 0.3 to 1.0 THz frequency range. The dashed vertical line indicates the *T_c_* = 84 K.

Figure 3a–c show 2D contour plots of THz signals at various temperatures for RCP, HLP and LCP THz incidences. Below 84 K, the time‐domain waveforms of all THz pulses shift forward, indicative of the superconducting transition. The corresponding THz spectra exhibit a blueshift (Figure S6 and Note S6). Figure 3d–f show the temperature‐dependent EMI SE of EBCO across the tested frequency range under RCP, HLP, and LCP THz incidences. In the normal state, EMI SE values for all frequencies are nearly uniform. In contrast, the superconducting state exhibits a universal increase in THz EMI shielding for all frequencies with decreasing temperature, with the SE enhancement becoming more pronounced at lower frequencies. This behavior is consistent with the temperature‐dependent conductive properties of EBCO.

Figure 3g shows the normalized transmittance $E_{\mathrm{peak}}\left(T\right)/E_{\mathrm{peak}}^{\mathrm{RT}}$, where *E*_Peak_(*T*) and $E_{\mathrm{peak}}^{\mathrm{RT}}$ are the peak values of the *E*_THz_ measured at temperature *T* and room temperature, respectively. $E_{\mathrm{peak}}\left(T\right)/E_{\mathrm{peak}}^{\mathrm{RT}}$ decreases gradually with cooling, reflecting enhanced THz conductivity of the EBCO film. Its rate of decrease accelerates below 84 K, a hallmark of the superconducting. At 20 K, $\;E_{\mathrm{peak}}\left(T\right)/E_{\mathrm{peak}}^{\mathrm{RT}}$ reaches 14% for RCP, LCP and HLP THz waves, demonstrating the EBCO film's robust THz shielding efficiency for all polarizations. Figure 3h plots the peak time shift $\Delta t\;\left(T\right)=t_{\mathrm{peak}}\;\left(T\right)-t_{\mathrm{peak}}^{\mathrm{RT}}$ as a function of temperature, where *t*_peak_(*T*) and $t_{\mathrm{peak}}^{\mathrm{RT}}$ are the peak amplitude time position measured at *T* and room temperature, respectively. Below *T_c_*, Δ*t*(*T*) decreases at a much steeper rate than the $E_{\mathrm{peak}}\left(T\right)/E_{\mathrm{peak}}^{\mathrm{RT}}$, making it a clear signature of the superconducting transition. Notably, both $E_{\mathrm{peak}}\left(T\right)/E_{\mathrm{peak}}^{\mathrm{RT}}$ and Δ*t*(*T*) for the MgO substrate are temperature independent (Figure S7 and Note S7). Figure 3i compares the average EMI SE values of the EBCO film over the 0.3 – 1.0 THz range as a function of temperature EMI SE (*T*) for different polarized THz waves. EMI SE (*T*) is found to be nearly independent of circular and linear THz polarization. This demonstrates that EBCO possesses robust THz EMI shielding performance, insensitive to the polarization state of incident THz radiation.

$E_{\mathrm{peak}}\left(T\right)/E_{\mathrm{peak}}^{\mathrm{RT}}$, Δ*t*(*T*) and EMI SE (*T*) show consistent behaviors under RCP, LCP and HLP THz incidence, indicating that the EBCO film hosts *d*‐wave superconductivity with preserved TRS. Notably, the orthorhombic crystal structure of EBCO introduces birefringence between horizontally and vertically polarized THz waves due to Cu─O chains aligned along the crystallographic *b*‐axis, which may induce weak time‐reversal symmetry breaking (TRSB). Nevertheless, the present work is impossible to distinguish weak TRSB signals originating from the film surface and bulk. Future work will integrate EBCO superconducting film with metamaterial architectures to further explore the order parameter symmetry of weak TRSB [73, 74, 75, 76, 77].

### 2.3 Ultrafast THz Modulation in EBCO Film Under Non‐Equilibrium State

**FIGURE 4:**
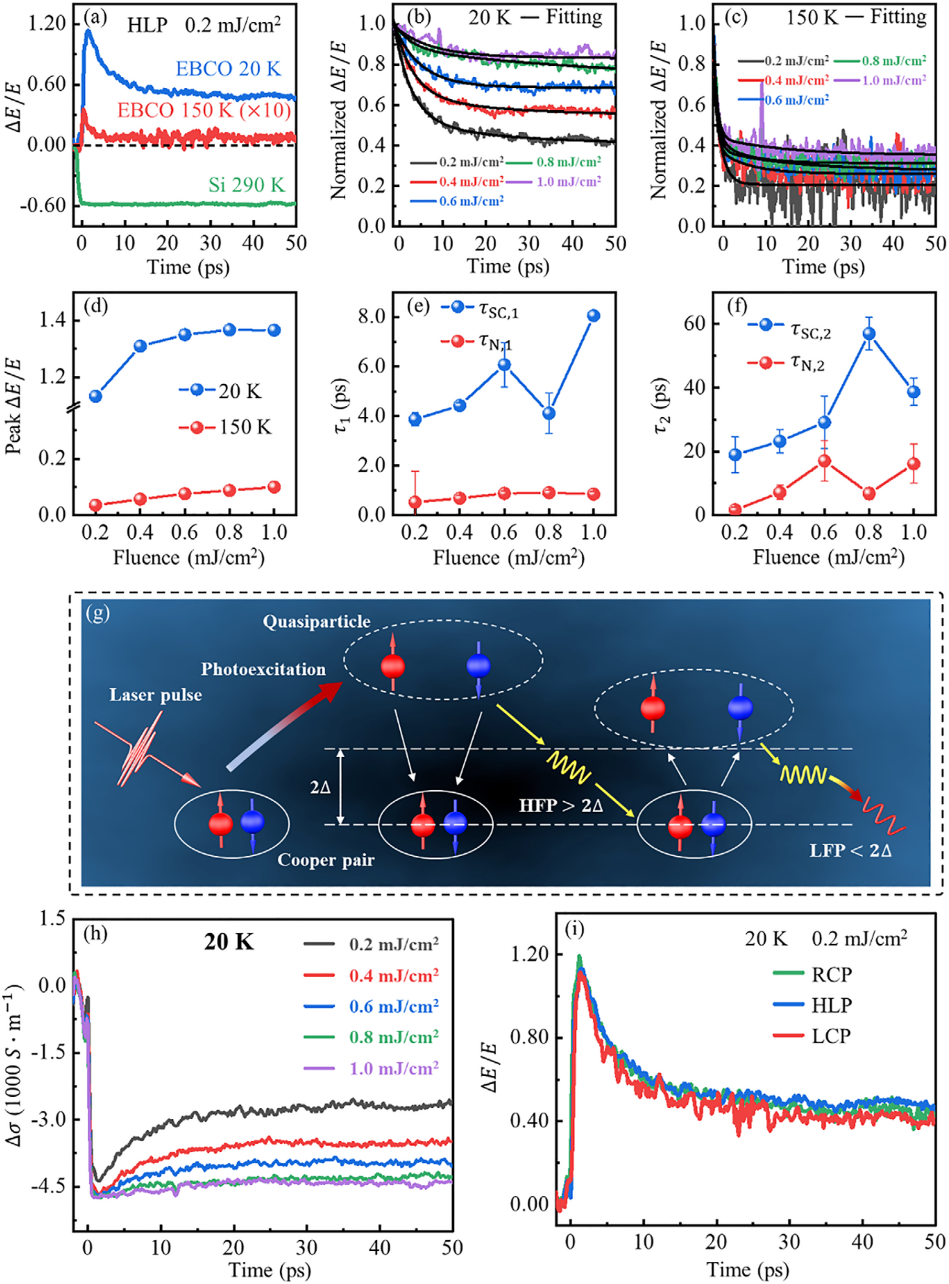
Pump‐fluence dependence of THz transmission Δ*E*(*t*)/*E* of EBCO. (a) Photoinduced transient HLP THz transmission Δ*E*(*t*)/*E* for the 43 nm thick EBCO film (measured at 150 and 20 K) and 0.5 mm‐thick Si (measured at 290 K), under 800 nm optical excitation with a pump fluence of 0.2 mJ/cm^2^. (b) The normalized Δ*E*(*t*)/*E* of EBCO measured at 20 K and (c) 150 K under different pump fluence. (d) Modulation amplitude (peak of Δ*E*(*t*)/*E*) as a function of pump fluence at 20 and 150 K. (e) The exponential relaxation time τ_1_ and (f) τ_2_ as a function of pump fluence at 20 and 150 K. The error bar represents the uncertainty in the fitting of the normalized Δ*E*(*t*)/*E*. (g) Schematic of photoinduced quasiparticle relaxation process. (h) The photoconductivity dynamics Δσ(*t*) of EBCO with different pump fluence measured at 20 K. (i) Δ*E*(*t*)/*E* of EBCO film measured for RCP, HLP and LCP THz waves at 20 K, under a pump fluence of 0.2 mJ/cm^2^.

Figure 4a compares the photoinduced transient THz transmission Δ*E*(*t*)/*E* of the EBCO film (*T* = 150 K and 20 K) and a 0.5 mm‐thick Si (*T* = 290 K) for the HLP THz pulse. Δ*E*  = *E*_pump_  − *E*, with *E* and *E*_pump_ denoting the transmitted THz pulse amplitude without and with photoexcitation, respectively. Under 800 nm optical excitation, EBCO film exhibits a positive Δ*E*(*t*)/*E* at all measured temperatures, in contrast to the negative Δ*E*(*t*)/*E* for Si. This opposite signal polarity indicates photo‐induced conductivity reduction in EBCO and conductivity increase in Si.

Figure 4b,c show the pump fluence dependent normalized Δ*E*(*t*)/*E* of EBCO at 20 and 150 K, respectively. The pump fluence dependent Δ*E*(*t*)/*E* for EBCO under RCP, HLP and LCP THz incidences are provided in Note S8 (Figure S8). Figure 4d plots the peak of Δ*E*(*t*)/*E* as a function of pump fluence at 20 and 150 K. We defined the modulation amplitude as [Δ*E*(*t*)/*E*]_peak_ × 100%, where [Δ*E*(*t*)/*E*]_peak_ is the peak value of Δ*E*(*t*)/*E*. It should be noted that the modulation amplitude in this work corresponds to the variation of the THz time‐domain signal peak, rather than the modulation amplitude at a specific frequency. Notably, at 20 K, EBCO delivers a large modulation amplitude, it reaches 113% at a pump fluence of 0.2 mJ/cm^2^, and further rises to 137% at 1.0 mJ/cm^2^. To quantify the modulation speed, we fit the normalized Δ*E*(*t*)/*E* with biexponential decay function [23]:

(5) $$\frac{\Delta E}{E}\;\left(t\right)=A_{1}\;\exp\left[\frac{-\left(t-t_{0}\right)}{\tau_{1}}\right]+A_{2}\exp\left[\frac{-\left(t-t_{0}\right)}{\tau_{2}}\right]+B$$

where τ_1_ and τ_2_ denote the relaxation time of fast and slow decay component, respectively. *A*_1_ and *A*_2_ are the amplitude of fast and slow decay component, respectively. *t*_0_ is the zero‐delay time at the peak of Δ*E*(*t*)/*E*, and *B* represents the time‐independent offset.

Figure 4e shows the pump fluence dependence of fast relaxation times τ_N,1_ (normal state) and τ_SC,1_ (superconducting state). Figure 4f shows the pump‐fluence dependence of slow relaxation times τ_N,2_ (normal state) and τ_SC,2_ (superconducting state). For practical device applications, the rate of THz modulation during the relaxation of the photoinduced nonequilibrium state to equilibrium is governed by the slow relaxation time constant τ_2_. Accordingly, we define τ_2_ as the characteristic modulation speed of our device. At 150 K, the modulation speed of EBCO film is τ_N,2_ = 1.7 ps, with a modulation amplitude is ∼3.5%. At 20 K, the modulation speed slows to τ_SC,2_ = 19.0 ps, while the modulation amplitude increases by a factor of 30 compared to the normal state. This highlights the potential of EBCO film as a high‐performance candidate for low temperature ultrafast THz modulator. Due to no Cooper pairs exist in the normal state, the negative THz photoconductivity observed in EBCO film originates from the photoinduced enhancement of hot carrier scattering rates. Upon absorption of the laser pulse by the EBCO film, photon energy transfers to the electron gas and increases the electron temperature. This raises the scattering rate, reduces the conductivity, and thereby enhances THz transmittance. The negative THz photoconductivity has been observed in several semimetallic materials, such as PdTe_2_, Co_2_MnSn and PtTe_2_ [12, 78, 79]. In the normal state, the two relaxation time constants τ_N,1_ and τ_N,2_ correspond to the electron‐phonon interaction and the lattice thermal relaxation process in EBCO, respectively. τ_N,1_ is nearly independent of pump fluence, with an average value of ${\bar{\tau }}_{N,1}\approx 0.77$ ps. τ_N,2_ increases from 1.66 to 16.15 ps, with rising pump fluence going from 0.2 mJ/cm^2^ to 1.0 mJ/cm^2^.

Quasiparticles dynamics in the superconducting state can be described by the Rothwarf‐Taylor model [80, 81]:

(6) $$\begin{array}{c} \begin{array}{c} \frac{d}{dt}n=-Rn^{2}+\eta N+I_{0}\\ \frac{d}{dt}N=-\frac{\eta N}{2}-\gamma \left(N-N_{T}\right)+\frac{Rn^{2}}{2}+J_{0} \end{array} \end{array}$$

where, *n* and *N* denote the densities of quasiparticles and high‐frequency phonons (HFPs), respectively. *R* is the quasiparticle recombination rate through HFPs emission. η is the probability of Cooper pair breaking via HFPs absorption. *N_T_* represents the thermally equilibrated HFPs density at temperature *T*, with γ as its decay rate. *I*_0_ and *J*_0_ are the external generation sources of quasiparticles and HFPs, respectively.

In Figure 4g, photoexcitation of cuprate HTS with laser pulses of photon energy exceeding the superconducting gap dissociates Cooper pairs into quasiparticles on a sub‐picosecond timescale [23], corresponding to *I*_0_. This sharp reduction in Cooper pair density leads to a drastic drop in conductivity, thereby increasing Δ*E*(*t*)/*E*. The photoconductivity Δσ in the superconducting state are derived from Δ*E*(*t*)/*E*, as shown in Figure 4h. The relaxation process via the quasiparticles release energy on picosecond time scale via scattering with remnant Cooper pairs, described by the − *Rn* ^2^ in Equation 6. Quasiparticles migrate to the band edge of the superconducting gap on a picosecond timescale, forming Cooper pairs and concomitantly emitting HFPs. Since HFPs energy exceed the superconducting gap, HFPs can further break Cooper pairs and regenerate quasiparticles, described by the η*N* in Equation 6. Quasiparticle generation from Cooper pairs persists as long as phonon modes with energy > 2Δ are populated. This process is eventually terminated either by anharmonic decay into low‐frequency phonons (LHPs) with energy < 2Δ or by phonons escape from the photoexcited region.

The fast relaxation time constant τ_SC,1_ originates from Cooper pair formation mediated by electron‐phonon interactions among hot quasiparticles, whereas the slow relaxation time constant τ_SC,2_ corresponds to the Cooper pair formation governed by 2Δ phonons. Figure 4e shows that τ_SC,1_ increases from ～3.87 ps at 0.2 mJ/cm^2^ to ～8.06 ps at 1.0 mJ/cm^2^. Figure 4f illustrates that τ_SC,2_ increases from ～18.96 ps to ～38.70 ps over the identical fluence range. At low pump fluences, only a relatively small fraction of Cooper pairs is excited into quasiparticles, thus the quasiparticles relax in the presence of a relatively large population of remnant Cooper pairs. In contrast, at high pump fluences, a large fraction of Cooper pairs is dissociated into quasiparticles, leaving an insufficient remnant Cooper pairs to interact with the quasiparticles. At a pump fluence of 0.2 mJ/cm^2^, the temperature rise of the film reaches ～93 K (see **Experimental Section**), resulting in substantial dissociation of Cooper pairs. Consequently, the scattering rate between quasiparticles and Cooper pairs is reduced, which causes a longer relaxation time constant. In both the normal and superconducting states, the component *B* increases with rising pump fluence (see in Figures S9 and S10), which is associated swith a large phonon population. The film returns to its initial temperature (and conductivity) as phonons dissipate from the film to the substrate via thermal transport on a nanosecond timescale.

To further investigate the ultrafast THz modulation of distinct polarized THz waves, we measured the Δ*E*(*t*)/*E* of the EBCO film at 20 K under RCP, HLP and LCP THz radiations, as presented in Figure 4i. LCP and RCP THz OPTP signals yielded nearly identical Δ*E*(*t*)/*E* profiles, with a chiral‐insensitive modulation amplitude of 113% at 20 K. The pump fluence dependences of τ_N,1_, τ_N,2_ and *B*_N_ for different polarized THz waves at 150 K are present in Note S9 (Figure S9). The pump fluence dependences of τ_SC,1_, τ_SC,2_ and *B*_N_ for different polarized THz waves at 20 K are present in Note S10 (Figure S10). At different pump fluence, we observed the almost identical Δ*E*(*t*)/*E* dynamics for RCP and LCP THz waves. The nearly identical dynamics of photoexcited quasiparticles and equilibrium electrodynamics responses in EBCO film, obtained with differently polarized THz waves, suggest that preserved TRS in the measured EBCO superconducting film is consistent with the absence of long‐range magnetic order in EBCO. In EBCO, Eu ions exist in the trivalent state with a 4f^6^ electronic configuration. Hund's rule predicts an effective Bohr magneton of 0 μ_*B*_ [82]. However, temperature‐independent magnetic susceptibility has been experimentally observed in Eu^3+^ ‐containing compounds (such as Eu_2_O_3_, EuF_3_, EuBO_3_) [83], which is attributed to Van Vleck paramagnetism arising from small energy separation between the ground and excited states. The experimentally measured magnetic moment of Eu^3+^ ion is ∼3.4 μ_*B*_ [82, 83], and temperature‐independent magnetic susceptibility has also been reported for EBCO [84], confirming the validity of Van Vleck paramagnetism in this material. Additionally, neither Mössbauer spectroscopy nor specific heat measurements have detected long‐range magnetic order in EBCO [85, 86], which explains the conservation of TRS observed in our study.

Finally, we would comment that the ultrafast THz modulation typically falls into two categories: absorption and bleaching, corresponding respectively to photoinduced reduction and enhancement of THz transmittance. Table 2 compares the key performance metrics of our EBCO film with those of other reported photoactive THz modulators, revealing EBCO's distinct advantages in both modulation amplitude and modulation speed. On the other hand, the EBCO film exhibits high THz EMI SE in the superconducting state, markedly suppressing THz wave transmission and thus enabling its designation as the logical “0” state under dark conditions (no optical excitation). By virtue of its ultra‐high modulation amplitude, EBCO shows THz transmittance enhancement under optical excitation, corresponding to the logical “1” state. Unambiguous switching between these two well‐defined states ensures high signal discrimination, highlighting its potential for photo‐driven logic gates and THz information processing devices. Furthermore, the integration of superconducting thin films with metasurface or metamaterial architectures open up new avenues for application in dual‐channel photonic switch, Josephson plasmon resonance, phase controllable THz frequency conversion and high‐temperature superinductor [23, 87, 88, 89]. Such hybrid composite structures combine the intrinsic ultrafast dynamic properties of superconductors with artificial electromagnetic field confinement effects. They are capable of boosting THz modulation efficiency and modulation speed, enabling multi‐functional resonant tuning and actively reconfigurable wavefront manipulation [90, 91].

**TABLE 2:** Comparison of modulation amplitude and speed for various materials.

Materials	Modulation type	Modulation amplitude @ pump fluence	Modulation speed (ps)	Operating temperature (K)	Refs.
Te	Absorption	65.3% (260 µJ/cm^2^)	9.95	300	[6]
Ge/Te	Absorption	87.6% (260 µJ/cm^2^)	9.25	300	[6]
HgTe	Absorption	73% (21 µJ/cm^2^)	14	300	[7]
HgTe	Absorption	81% (7 µJ/cm^2^)	9	5	[7]
GaAs	Absorption	75% (127 µJ/cm^2^)	370	300	[92]
Cd_3_As_2_	Absorption	63% (63.5 µJ/cm^2^)	15.5	300	[93]
Ge	Absorption	25% (2250 µJ/cm^2^)	7	300	[94]
FA_0.4_MA_0.6_PbI_3_/Meta	Absorption	16.4% (12.7 µJ/cm^2^ @0.44 THz)	3.5	300	[95]
MAPbI_3_/Meta	Absorption	23% (7 µJ/cm^2^ @0.7 THz)	6	300	[95]
2D perovskites/Meta	Absorption	13.7% (50 µJ/cm^2^ @0.88 THz)	20	300	[96]
MoS_2_	Absorption	100% (254 µJ/cm^2^ @0.82 THz)	143	300	[97]
Ge‐based metamaterials	Absorption	22.68% (153 µJ/cm^2^)	17	300	[98]
Si	Absorption	58% (200 µJ/cm^2^)	ms	300	This work
PdTe_2_	Bleaching	10% (1130 µJ/cm^2^)	529.45	300	[12]
Co_2_MnSn	Bleaching	12% (760 µJ/cm^2^)	181	300	[78]
Fe_4_GeTe_2_	Bleaching	∼1% (560 µJ/cm^2^)	∼100	300	[99]
PtTe_2_	Bleaching	12% (542 µJ/cm^2^)	ns	300	[79]
YBa_2_Cu_3_O_7_‐based metamaterials	Bleaching	44% (254.8 µJ/cm^2^ @0.4 THz)	<40	5	[23]
YBa_2_Cu_3_O_7_‐based metamaterials	Bleaching	76% (52 µJ/cm^2^ @0.6 THz)	23.1	7	[89]
EuBa_2_Cu_3_O_7_	Bleaching	113% (200 µJ/cm^2^)	19.0	20	This work
EuBa_2_Cu_3_O_7_	Bleaching	3.5% (200 µJ/cm^2^)	1.7	150	This work

## 3 Conclusion

In summary, we show that EBCO films exhibit high‐efficiency THz EMI shielding performance, originating from the ultrahigh intrinsic THz conductivity of their superconducting state. Furthermore, we realize ultra‐efficient photoinduced THz transmission modulation in EBCO, driven by a significant reduction in Cooper pair density and the ultrashort relaxation lifetime of Cooper pairs – quasiparticle recombination. In addition, chiral‐insensitive THz responses under both photoexcited and non‐photoexcited conditions in the superconducting state suggest the preservation of TRS or weak TRSB in EBCO. Notably, EBCO films are promising for high‐performance THz EMI shielding and ultrafast THz optical switching. They hold potential for next‐generation THz photonic devices, as well as cryogenic quantum computing and high‐fidelity quantum information transmission, where low‐temperature THz wave manipulation is essential.

## 4 Experimental Section

### 4.1 Polarization Controllable THz Waves Emission

The SMTE consisted of ferromagnetic/nonmagnetic heterostructure integrated with strip‐patterned metasurface. A *z*‐axis‐propagating laser pulse irradiated the SMTE to generate THz pulses. As shown in Figure 1a, THz wave polarization was characterized by measuring the *E_x_* and *E_y_* components using a pair of THz polarizers. The primary THz generation mechanism in SMTE was the inverse spin Hall effect [100]. In addition, orbital‐to‐charge current conversion had also been demonstrated to contributing to THz radiation in such heterostructure [101]. Laser excitation induced a z‐axis spin current *j_s_* in Fe layer, which converts to a charge current *j_c_* =  γ*j_s_* × *M*/|*M*| in both Pt and W layers. According to Maxwell's equations, *j_c_* acts as an electric dipole emitting THz radiation, *E*_THz_(*t*)∝∂*j_c_*/∂*t*, perpendicular to the in‐plane magnetic field. The picosecond‐scale *j_c_* confined the radiation into the THz frequency range. The stripe‐patterned metasurface caused charge accumulation at the edges of the stripes due to the *j_y_* component of *j_c_*, generating an electric potential that induces a π/2 phase difference between *j_y_* and *j_x_* [54]. Rotating the magnetic field angle θ_H_ modulates *E_x_* and *E_y_* amplitudes, yielding RCP, HLP, and LCP THz waves at θ_H_ = − 60°, 0°, and 60°, respectively.

### 4.2 Optical Pump THz Emission and Modulation Spectroscopy

A Ti: sapphire laser amplifier produced 35 fs pulse width, 800 nm central wavelength laser pulses at 1 kHz repetition rate. A beam splitter split the laser pulse into two beams (9:1 ratio). The high‐power beam served as pump pulse and the low‐power one as probe pulse. The pump pulse was then further split equally into two beams, denoted as pump pulse 1 and pump pulse 2. Pump pulse 1 excited the SMTE to generate THz waves, with the SMTE magnetized to saturation by a 1250 Gs magnetic field in the *x‐y* plane. Rotating the magnetic field yielded RCP (θ_*H*_ = − 60°), LCP (θ_*H*_ = + 60°), and HLP (θ_*H*_ = 0°) polarized THz waves. Off‐axis parabolic mirrors collected and focused the THz waves onto the EBCO film. After transmitting through the EBCO, another mirror pair collinearly focused the THz waves with the probe beam onto a 1.0 mm thick ZnTe <110> crystal. The probe pulse detected the THz signal via free‐space electro‐optical sampling. Switching pump pulse 2 off/on enabled selective probing of equilibrium and non‐equilibrium Cooper pair electrodynamics in EBCO, respectively. The sample was positioned at the THz focal point, with a 5 mm‐diameter aperture used in the measurement. This aperture size is significantly larger than the THz detection spot (～2.5 mm in diameter), to minimize the diffraction‐induced errors during the experimental process. A continuous flow liquid helium cryostat (Oxford SpectromagPT) was used for cryogenic measurements, with temperature‐dependent ultrafast THz signals recorded from 20 to 300 K.

### 4.3 Sample Preparation

A 43 nm‐thick EBCO film was deposited on a 0.5 mm‐thick single‐crystalline MgO (001) substrate via pulsed laser deposition. Prior to growth, the substrate was heated to 750 °C under an oxygen partial pressure of 50 mTorr. The deposition was performed with a laser energy of 300 mJ and 1 Hz repetition rate. The film thickness was characterized by x‐ray reflectivity (XRR) measurement. Superconductivity of the EBCO film was confirmed using four‐probe resistance measurements. We used the same MgO substrate that grows the EBCO film as the THz reference.

### 4.4 Estimation of EBCO Film Heating Temperature

We estimate the temperature rise of film by Δ*T*  =  *u*/*C_v_* [102], where *u* = 4.65 × 10^7^ J/m^3^ was the laser energy absorbed per unit volume with a pump fluence of 0.2 mJ/cm^2^, and *C_v_* ～3×10^5^ J/(m^3^∙K) was the volumetric specific heat capacity of the film at 20 K [103]. Considering that reflectance of EBCO was negligible and its transmittance is ∼60% at 800 nm at 20 K [104], we estimate the temperature rise of ∼93 K for the EBCO thin film at an initial temperature of 20 K under a pump fluence of 0.2 mJ/cm^2^. It should be noted that this estimation ignores heat diffusion from the film to the substrate and assumes all non‐ transmitted energy was absorbed, thus providing an upper‐bound estimate. This elevated temperature far exceeds the *T_c_* of EBCO (84 K), and thus the superconducting state was significantly disrupted.

## Author Contributions

**Zhangshun Li**: investigation, formal analysis, validation, visualization, writing – original draft, writing – review and editing. **Meng Yang**: investigation, validation, formal analysis, visualization. **Zuanming Jin**: conceptualization, methodology, data curation, supervision, resources, formal analysis, project administration, validation, investigation, funding acquisition, visualization, writing – review and editing, writing – original draft. **Junxue Li**: conceptualization, investigation, methodology, validation, visualization, writing – review and editing, writing – original draft, funding acquisition, project administration, supervision, resources, formal analysis. **Zhiqiang Lan**: investigation, validation, formal analysis. **Chen Huang**: investigation, validation, formal analysis. **Alexei V. Balakin**: visualization, validation, methodology, writing – review and editing. **Alexander P. Shkurinov**: methodology, validation, visualization, writing – review and editing. **Yan Peng**: resources, supervision, project administration, writing – review and editing, funding acquisition, writing – original draft. **Yiming Zhu**: funding acquisition, writing – original draft, writing – review and editing, project administration, resources, supervision. **Songlin Zhuang**: funding acquisition, writing – original draft, writing – review and editing, project administration, resources.

## Conflicts of Interest

The authors declare no conflicts of interest.

## Supporting information




**Supporting File**: advs77616‐sup‐0001‐SuppMat.docx.

## Data Availability

The data that support the findings of this study are available from the corresponding author upon request.
