# Supplementary material for: Dual‐Functional Terahertz Manipulation of EuBa2Cu3O7 Superconductors via Cooper Pair Dynamics
Source: Adv Sci (Weinh). 2026 Sep 8:e77616. Online ahead of print. doi: 10.1002/advs.77616 (PMC13554402; doi:10.1002/advs.77616)
Supplement: Supplementary file 1 [file ADVS-9999-e77616-s001.docx]

Supplemental Information for

Dual-Functional Terahertz Manipulation of EuBa_2_Cu_3_O_7_ Superconductors via Cooper Pair Dynamics

*Zhangshun Li^1^, Meng Yang^2^, Zuanming Jin^1,5,*^, Junxue Li^2,*^, Zhiqiang Lan^1^, Chen Huang^1^, Alexei V. Balakin^3,4^, Alexander P. Shkurinov^3,4^, Yan Peng^1,5,*^, Yiming Zhu^1,5,*^, and Songlin Zhuang^1^*

^1^National Key Laboratory of Terahertz Perception and Communication (TPCL), THz Technology Innovation Research Institute, THz Spectrum and Imaging Technology Cooperative Innovation Center, Shanghai Key Lab of Modern Optical System, University of Shanghai for Science and Technology, Shanghai 200093, China

^2^Department of Physics, Southern University of Science and Technology, Shenzhen 518055, China

^3^Faculty of Physics, Lomonosov Moscow State University, Leninskie Gory 1, Moscow 19991, Russia

^4^Branch “ILIT-Shatura” of KCCP of NRC “Kurchatov Institute,” Svyatoozerskaya 1, Shatura, Moscow Region 140700, Russia

^5^Shanghai Institute of Intelligent Science and Technology, Tongji University, Shanghai 200092, China

Zhangshun Li, Meng Yang and Zuanming Jin contributed equally to this work.

E-mail: Zuanming Jin (physics_jzm@usst.edu.cn), Junxue Li (lijx3@sustech.edu.cn), Yan Peng (py@usst.edu.cn), Yiming Zhu (ymzhu@usst.edu.cn)

**Supplementary Note 1: Dynamic range of SMTE**

The peak electric field strength $E_{\mathrm{THz}}^{\mathrm{Peak}}$ of SMTE is approximately 378 kV/cm. In addition, the SMTE achieves a maximum dynamic range of ~51 dB at 0.4 THz.

**Figure S1.** (a) THz time-domain signal and (b) dynamic range of SMTE

**Supplementary Note 2: THz-TDS signals of EBCO at different temperatures**

Figure S2(a)-S2(c) show the time domain signals $E_{\mathrm{THz}}(t)$ of EBCO measured with RCP, HLP and LCP THz waves at temperature between 20 K and 300 K. The dashed line represents the peak position of $E_{\mathrm{THz}}(t)$ at 300 K. As the temperature decreases, a clear time shift and decrease in $E_{\mathrm{THz}}(t)$ are observed.

**Figure S2.** Time domain signals $E_{\mathrm{THz}}(t)$ of EBCO measured with (a) RCP, (b) HLP and (c) LCP THz waves at different temperatures.

**Supplementary Note 3:** **EMI SE values of EBCO measured using RCL and LCP THz pulses**

**Figure S3.** EMI SE values of EBCO measured using RCL (a) and LCP (b) THz pulses.

**Supplementary Note 4:** **THz conductivity spectra with different polarization THz waves**

Figure S4(a)-S4(c) show the real part of THz conductivity $\sigma_{\mathrm{Re}}$ measured with RCP, HLP and LCP THz waves at temperature between 20 K and 150 K. The imaginary part of THz conductivity $\sigma_{\mathrm{Im}}$ as shown in Figure S4(d)-S4(e).

**Figure S4.** The real part of THz conductivity $\sigma_{\mathrm{Re}}$ measured with (a) RCP, (b) HLP and (c) LCP THz waves at different temperatures. The imaginary part of THz conductivity $\sigma_{\mathrm{Im}}$ measured with (d) RCP, (e) HLP and (f) LCP THz waves at different temperatures.

**Supplementary Note 5: Fitting of THz conductivity measured via HLP THz waves across the temperature range of 20 K to 150 K**

**Figure S5.** (a-e) Fitting of THz conductivity via two-fluid mode at 20 K to 60 K, and (f-g) via Drude-Smith mode at 100 K to 150 K.

**Supplementary Note 6: Normalized 2D contour plots of THz spectra for RCP, HLP and LCP THz waves**

**Figure S6.** Normalized 2D contour plots of THz spectra at selected temperatures for RCP (a), HLP (b) and LCP (c) polarizations

**Supplementary Note 7: THz-TDS signals of MgO substrate at different temperatures**

Figure S7(a)-S7(c) show the time domain signals $E_{\mathrm{THz}}(t)$ of MgO substrate measured with RCP, HLP and LCP THz waves between 20 K and 300 K. The dashed line represents the peak position of $E_{\mathrm{THz}}(t)$ at 300 K. Figure S7(d)-S7(e) present the transmittance $E_{p\mathrm{eak}}(T)/E_{\mathrm{peak}}^{\mathrm{RT}}$ and time shift $\Delta t$ as a function of temperature for RCP, HLP and LCP THz waves. Both $E_{p\mathrm{eak}}(T)/E_{\mathrm{peak}}^{\mathrm{RT}}$ and $\Delta t$ remain nearly temperature independence across the entire temperature range for RCP, HLP and LCP THz waves.

**Figure S7.** Time domain signals $E_{\mathrm{THz}}(t)$ of MgO substrate measured with (a) RCP, (b) HLP and (c) LCP THz waves at different temperatures. (d) Peak transmittance $E_{p\mathrm{eak}}(T)/E_{\mathrm{peak}}^{\mathrm{RT}}$ and (h) peak time shift $\Delta t$ as a function of temperature.

**Supplementary Note 8: Transient THz transmission at different pump fluence**

Figure S8(a)-S8(c) show the photoinduced transient THz transmission $\Delta E(t)/E$ of EBCO at 150 K, probed with RCP, HLP and LCP THz waves at different pump fluence. The corresponding measurements at 20 K are shown in Figure S8(d)-S8(f). No significant difference in $\Delta E(t)/E$ is observed probed with RCP, HLP and LCP THz waves under identical conditions.

**Figure S8.** The $\Delta E(t)/E$ of EBCO measured at 150 K for (a) RCP, (b) HLP and (c) LCP THz waves under different pump fluence. The $\Delta E(t)/E$ of EBCO measured at 20 K for (d) RCP, (e) HLP and (f) LCP THz waves under different pump fluence.

**Supplementary Note 9: Transient THz transmission with different polarized THz waves at 150 K**

Figure S9(a) shows the $\Delta E(t)/E$ of EBCO probed with RCP, HLP and LCP THz waves at 150 K. Figure S9(b)-S9(d) show the temperature dependence of $B_{N}$, $\tau_{N,1}$ and $\tau_{N,2}$ for different polarized THz waves at 150 K. The average values of $\tau_{N,1}$ measured by LCP, RCP, and HLP THz waves are pump fluence independent, 0.77, 0.75, and 0.67 ps, respectively.

**Figure S9.** (a) $\Delta E(t)/E$ of EBCO thin film measured with different polarized THz waves at 150 K, under a pump fluence of 0.2 mJ/cm^2^. (b) The offset $B_{N}$, (c) the fast time constant $\tau_{N,1}$ and (d) slow time constant $\tau_{N,2}$ as a function of pump fluence at 150 K.

**Supplementary Note 10: Transient THz transmission with different polarized THz waves at 20 K**

Figure S10(a)-S10(c) show the temperature dependence of $B_{\mathrm{SC}}$, $\tau_{SC,1}$ and $\tau_{SC,2}$ for different polarized THz waves at 20 K.

**Figure S10.** (b) The offset $B_{\mathrm{SC}}$, (c) the fast time constant $\tau_{SC,1}$ and (d) slow time constant $\tau_{SC,2}$ as a function of pump fluence at 20 K.
